# Survival Time Discrepancy among Under-Five-Year Children of Rural Parts of Ethiopia

**DOI:** 10.34172/jrhs.2022.78

**Published:** 2022-02-03

**Authors:** Lema Abate, Samuel Getachew

**Affiliations:** ^1^Department of Statistics, College of Natural and Computational Sciences, Mizan-Tepi University, Ethiopia; ^2^Department of Biology, College of Natural and Computational Sciences, Mizan-Tepi University, Ethiopia

**Keywords:** Ethiopia, Risk Factors, Survival analysis, Under-five

## Abstract

**Background:** Ethiopia is ranked as the fifth of heavy under-five death burdened countries with the highest burden in its rural areas. This study aimed to identify the determinants of under-five deaths in rural parts of Ethiopia.

**Study Design:** A population-based cross-sectional study.

**Methods:** The data for this study was extracted from the 2016 Ethiopian Demographic and Health Survey. Descriptive analysis, non-parametric estimation, and Cox proportional hazards regression model were used to examine the determinants of under-five mortality.

**Results:** A total of 7301 under-five eligible children from rural areas were involved in this survey, and 6.5% of the cases were passed away before reaching their fifth birthday. Male children’s death accounted for 59.7% of the death rate in the participants. An estimated median survival time was 31 months [95% CI: 30-32]. About 83% of children’s death occurred among children delivered at home. Cox proportional hazard regression model revealed that gender, delivery-place, family-size, mother’s education, number of children, contraceptive use, and source of drinking water had significant effects on survival time of underfive children. Under-five mortality was significantly fewer in female children (HR = 0.728; 95% CI: 0.606- 0.875, *P* = 0.001), children delivered at health facilities (HR = 0.738; 95% CI: 0.572-0.951, *P* = 0.019), and those from secondary and above educated mothers (HR = 0.464; 95% CI: 0.301-0.714, *P* = 0.001), compared to the reference category.

**Conclusion:** Significant risk factors were associated with under-five mortality in rural areas. Delivering in health facilities, uses of contraceptives, mother’s education, and improvement of infrastructures should be areas of concern to decrease under-five children’s deaths.

## Background

 Under-five child death is the likelihood of dying between the period of delivery and the fifth birthday of children articulated per 1000 live births, and it is a good indicator of the level of child health and the overall development of countries.^1^ The global under-five death rate was reduced from 93 deaths per 1000 live births in 1990 to 41 in 2016.^2^ The 2016 Sustainable Development Goals had designed strategies and interventions that can significantly contribute to reducing under-five mortality rate below 25 per 1000 live births or less by 2030.^3^ A number of nations have reported reducing the under-five mortality; however, developing countries of sub-Saharan Africa still have a really high under-five death rate.^4^ Of an estimated 5.3 million under-five deaths in 2018, 52% of these deaths were reported from sub-Saharan Africa with an average under-five mortality of 78 per 1000 live births that could be attributed to several factors.^2,5-7^

 More recent studies from Bangladesh, Nigeria, South Sudan, and sub-Saharan Africa demonstrated residence area,^8,9^ family size,^10,11^ source of water,^11,12^and toilet facility as determinant factors of under-five mortality.^11,13^ Ethiopia is ranked as a fifth of heavy under-five deaths burdened countries of sub-Saharan and Southern Asia regions.^2^ The country showed a major improvement with a gradual decline from 244.8 in 1970 to 50.7 deaths per 1000 live births in 2019. This achievement was actually brought through improvements in overall socioeconomic conditions and wider area coverage of health facilities with good interventions in prevention and treatments of the major causes of child mortality.^14^ However, studies across the country implied the significant effect of the residency areas on the under-five deaths.^15,16^ Other studies that used the Ethiopian Demographic and Health Survey (EDHS) estimate of 2000, 2005, and 2011 also revealed the worst situation of child mortalities in rural parts of the country.^10,16^

 According to studies, antenatal and post-natal cares determine the survival of children with a greater risk of under-five deaths among children from mothers who have not attended the service. Gender of child and family head, household economic status, and mother’s education are also reported to significantly influence the survival rate of under-five children with variability and inconsistences across the countries.^8,15,17,18^

 However, identifying risk factors of under-five mortality and decreasing these disproportions will help to save more children’s lives through intervention mechanisms and inform the public health officials and policy designers to design strategies that accelerate the reduction of under-five child mortality^5^. Therefore, this study aimed to identify factors that determine the survival time of under-five children in rural parts of Ethiopia.

## Methods

###  Study settings

 According to the 2018 estimate, the total population of Ethiopia was above 108 million, making it the 12^th^ rapid population growth country in the world and the second-most populous country in Africa, following Nigeria. More than 80% of the total population lives in rural areas, and the country’s economy is predominantly agriculture-based. Ethiopia’s rapid population growth is putting the country under increasing pressure on land resources, an increase of land degradation and deforestation, and an increase in scarcity of basic necessities such as food.

 The data for this study was extracted from the 2016 EDHS, which was a population-based cross-sectional study collected between January 18, 2016, and June 27, 2016, throughout the country. This dataset is accessible online using the link received from the Demographic and Health Survey (DHS) database: https://www.dhsprogram.com/data/dataset_admin/login_main.cfm.

 According to EDHS of 2016, the samples were selected in two phases. In the first phase, 645 clusters (202 from urban and 443 from rural) were randomly selected proportional to the household extent from the sampling strata, and secondly, 28 households per cluster were selected using systematic random sampling, and only rural clusters were incorporated in this study.

 According to the EDHS, report data were collected using different questionnaires, and the data of the child mortality and associated factors were obtained from a questionnaire of women who met the eligibility criteria (women aged 15-49 years). From the samples of 18 008 households proposed, 16 650 households were interviewed for an individual interview, and 16 583 qualified women were identified from the interviewed household. Interviews with about 15 683 women aged 15-49 were completed,^19^ and 10 641 women were included due to having children preceding five years survey to compute the under-five mortality. Accordingly, 7301 children born in rural areas between 2011 and 2015 five years prior to the assessment were considered in this investigation.

###  Study variables

 Time to death of under-five children which was measured in months (0-59 months) five years before was the response variable of the study. The under-five child mortality was used as an event and coded as 1 if the child died and 0 if the child survived during the survey. Predictor variables included a place of delivery, gender of children, birth weight of children, gender and age of the head of the family, family size, mother’s age at first birth, marriage to first birth, current marital status, father’s and mother’s education, place of residence, religion, number of under-five children, wealth index, antenatal care (ANC) visit, contraceptive method use, toilet availability, and source of drinking water.

###  Statistical methods 

 All obtained data were cleaned, coded, and examined by SPSS software (version 20) and STATA statistical software (version 14). Descriptive analysis, non-parametric estimation, and Cox proportional hazard regression (PH) model were employed to examine the risk factors of under-five mortality. The Cox PH model is one of the common PH models, which is a broadly applicable, and the most widely used method of survival analysis.^20^ Predictor variables those found significant in the univariable analysis by considering a *P* value of 0.20-0.25, and more important variables that were insignificant in the univariable analysis were involved in the multivariable Cox PH regression analysis. Moreover, the estimated hazard ratios with a *P* value less than 5% were used to indicate the statistical significance of the variable in multivariable analysis. In addition, the log-rank test was used to check the association of survival times among the different groups of the explanatory variable.

###  Statistical tests of proportional hazards model assumptions

 The goodness of fit testing approach is appealing because it provides a test statistic and *P* value for assessing the PH assumption for given covariates of interest. Rho tells the relationship between time and residuals. When the test of correlation (rho) is insignificant, it indicates the proportional hazards assumption is fulfilled. Moreover, it is also possible to see its global test, and if it is greater than 0.05, the assumption has been satisfied by the covariates in the model. The scatter plots of Scaled Schoenfeld residuals were also used to check PH assumptions. If the PH assumption is met, Schoenfeld residuals should look horizontal since the scaled Schoenfeld residuals would be independent of survival time.

## Results

 Out of 7301 under-five children eligible for this investigation, 6.5% of the cases were passed away before reaching their fifth birthday, and 93.5% of the children were censored. An estimated median survival time of under-five children in the rural area was 31 months (95% CI: 30-32). Out of the total participants, almost half (51.4%) of them were male, and 59.7% of the cases died preceding the five years of the survey. More than three-fourths (77.2%) of the children were delivered at home, while the rest (22.8%) were delivered at health centers and other places. About 41% of the under-five children had been delivered with an average size, and the death proportions among those having larger than average size, average size, and smaller than average size were 25.2%, 40.3%, and 34.5%, respectively. However, the death proportion of children who were born at home was found exceedingly larger (83%) (Table 1).

**Table 1:** Descriptive summaries of under-five children mortality and associated risk factors in rural parts of Ethiopia in EDHS, 2016

**Variables**	**Death**		**Survival**		**Total**	
	**Number**	**Percent**	**Number**	**Percent**	**Number**	**Percent**
**Child related variables**						
Gender of the children						
Female	192	40.3	3358	49.2	3550	48.6
Male	284	59.7	3467	50.8	3751	51.4
Place of delivery						
Health center	74	15.5	1472	21.6	1546	21.2
Home	395	83.0	5243	76.8	5638	77.2
Other places	7	1.5	110	1.6	117	1.6
Weight of child at birth						
More than average	120	25.2	2021	29.6	2141	29.3
Average	192	40.3	2771	40.6	2963	40.6
Less than average	164	34.5	2033	29.8	2197	30.1
**Parental related variables**						
Gender of household head						
Male	404	84.9	5726	83.9	6130	84.0
Female	72	15.1	1099	16.1	1171	16.0
Age of household head						
Less than 30	119	25.0	1583	23.2	1702	23.3
30 and above	357	75.0	5242	76.8	5599	76.7
Age of mother at first birth						
19 and less	302	63.4	4473	65.5	4775	65.4
20 and above	174	36.6	2352	34.5	2526	34.6
Marriage to first birth						
Less than 2 year	294	61.8	4236	62.1	4530	62.0
2 year and above	182	38.2	2589	37.9	2771	38.0
Current marital status						
Single	4	1.7	84	1.2	92	1.3
Married	464	96.6	6487	95.1	6947	95.2
Others	8	1.7	254	3.7	262	3.5
Mother education						
No education	362	76.1	4929	72.2	5291	72.5
Primary	92	19.3	1608	23.6	1700	23.3
Secondary and above	22	4.6	288	4.2	310	4.2
Father education						
No education	253	53.1	3410	50.0	3663	50.1
Primary	85	29.0	1242	31.8	1327	18.2
Secondary and above	138	17.9	2173	18.2	2311	31.7
ANC visit						
Less than 4	395	83.0	5697	83.5	6092	83.4
4 and above	81	17.0	1128	16.5	1209	16.6
Contraceptive method use						
No	288	60.5	3785	55.5	4073	55.8
Yes	188	39.5	3040	44.5	3228	44.2
Household related variables						
Region						
Tigray	33	6.9	626	9.2	659	9.0
Afar	73	15.3	769	11.3	842	11.5
Amhara	39	8.2	720	10.5	759	10.5
Oromia	69	14.5	1212	17.8	1281	17.5
Somali	73	15.3	961	14.1	1034	14.2
Benishangul	50	10.5	632	9.3	682	9.3
SNNPR	58	12.2	956	14.0	1014	13.9
Gambela	32	6.7	396	5.8	428	5.9
Harari	30	6.3	320	4.7	350	4.8
Dire Dawa	19	4.0	233	3.4	252	3.5
Family size						
1-3	177	37.2	2772	40.6	2949	40.4
4 and above	299	62.8	4053	59.4	4352	59.6
Religion						
Orthodox	100	21.0	1738	25.5	1838	25.2
Protestant	70	14.7	1245	18.2	1315	18.0
Muslim	287	60.3	3663	53.7	3950	54.1
Other	19	4.0	179	2.6	198	2.7
Number of under-five children						
No	31	6.5	185	2.8	216	3.0
1-2	380	79.8	5183	75.9	5563	76.2
3 and above	65	13.7	1457	21.3	1522	20.8
Economic status						
Poor	333	70.0	4409	64.6	4742	65.0
Middle	65	13.6	1120	16.4	1185	16.2
Rich	78	26.4	1296	19.0	1374	18.8
Toilet availability						
No	346	72.7	4949	72.5	5295	72.5
Yes	130	27.3	1876	27.5	2006	27.5
Source of drinking water						
Piped	158	33.2	1996	29.2	2154	29.5
Others	318	66.8	4829	70.8	5147	70.5

 Approximately, 72% of mothers’ of the children in this study were uneducated, and 65.4% of them were in the age group of fewer than 20 years at their first delivery. The children’s death proportions from uneducated and less than 20-year-old mothers were 76% and 63.4%, respectively. Above 83% of the study children’s mother were enrolled in ANC during their pregnancy less than 4 times; however, the child’s mortality rate among these mothers were 83%. Moreover, 55.8% of the participants were from mothers that had not used contraceptive methods, and the death proportion was reported to be 60.5%. Most of the heads of the household were males (84%), and three-fourths of them were aged 30 years and above. About 53.1% and 17.9% of the children from uneducated fathers, as well as secondary and above-educated fathers, died before their fifth year’s birthday.

 The children participating in this study were from all regional states of Ethiopia; however, children from Oromia (17.5%), Somali (14.2%), South Nation Nationality and People Region (SNNPR) (13.9%), Afar (11.5%), and Amhara (10.5%) covered the largest proportion. Of these, the recorded death proportions of Afar, Somali, Oromia, SNNPR, and Benishangul Gumuz were 15.3%, 15.3%, 14.5%, 12.2%, and 10.5%, respectively. The majority of the mothers had 1-2 other under-five children preceding the five years survey, while 3% of them had no other child. Out of 7301 participants, 65% of the children were from poorer families, and the recorded death proportion was 70% within this wealth index category. Of all participants, 59.6% of the children were from families having four and above family size, while the remaining (40.4%) were from families having less than four family sizes. The death proportion among those having > 3 family was surprisingly large (62.8%) (Table 1).

**Figure 1:**
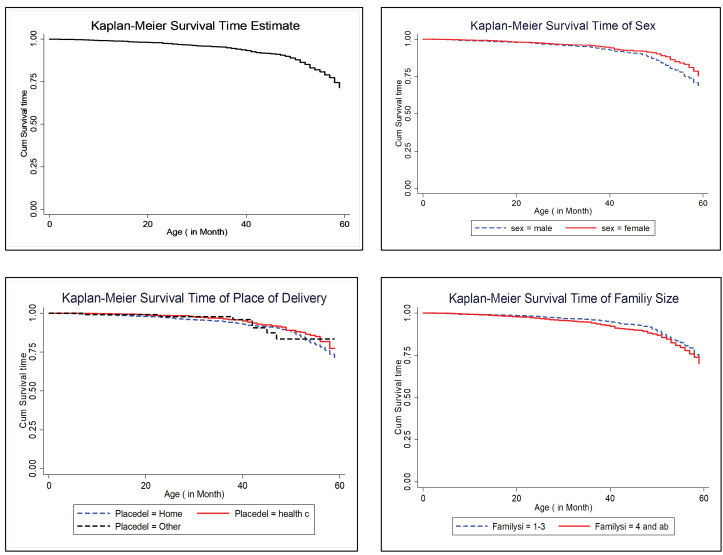
Kaplan-Meier estimate curves.

 Non-parametric methods in survival analysis are very important to visualize the survival time of patients under different groups of covariates; therefore, the Kaplan-Meier estimate curve and log-rank test were used to compare the survival rates of two or more groups of under-five children in rural parts of Ethiopia. Accordingly, gender of children, family size, place of residency, religion, educational background of the child’s father and mother, number of under-five children, wealth index, and contraceptive method use were statistically significant (Table 2). This result implied that the survival time of under-five children under the different categories of covariates had different survival times, and all covariates were checked by Kaplan-Meier estimate curves as some of them are put in Figure 1.

**Table 2:** Log-rank test of association among variables and survival time

**Variables**	**Log rank test**		
	**Chi-square**	**df**	***P*** ** value**
Gender of the children	11.730	1	0.001
Place of delivery	6.570	2	0.037
Weight of child at birth	4.539	2	0.103
Gender of household head	0.020	1	0.887
Age of household head	0.958	1	0.328
Family size	5.700	1	0.017
Age of mother at first birth	1.776	1	0.183
Marriage to first birth	0.030	1	0.863
Current marital status	1.709	2	0.426
Father education	2.895	2	0.035
Region	26.018	9	0.002
Mother education	5.436	2	0.046
Religion	18.238	3	0.000
Number of under-five children	9.250	2	0.010
Wealth index	7.934	2	0.019
ANC visit	0.083	1	0.773
Contraceptive method use	7.356	1	0.007
Toilet availability	0.015	1	0.901
Source of drinking water	4.335	1	0.037

Source: Ethiopian Demographic Health Survey, 2016.

**Figure 2:**
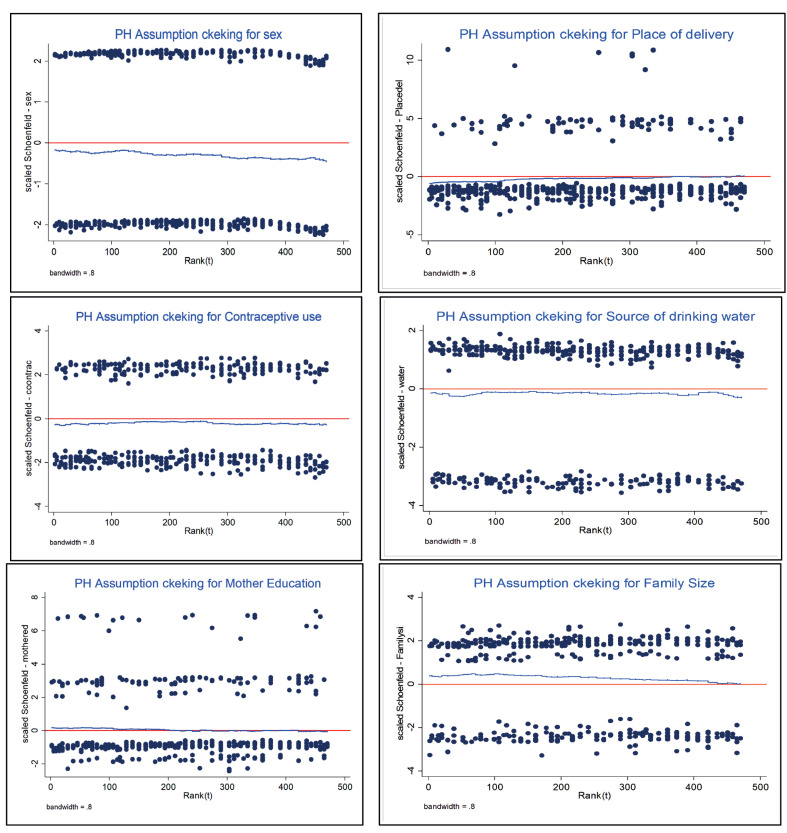
Test of proportional hazards assumption by Scaled Schoenfeld residuals.

 According to the results in Table 3 and Figure 2, the assumptions of the PH model were satisfied since the global test value is insignificant, and the Scaled Schoenfeld residuals plot was horizontal. Therefore, the PH model was used in this study to fit the under-five children data. To attain the final multivariable Cox PH model, the assumption of PH and the univariable analysis was conducted, and variables that were significant in the univariable analysis were involved in the final Cox PH model. Gender of children, place of delivery, family size, mother education, number of under-five children in the family, use of contraceptives, and source of drinking water had a statistically significant effect on the survival time of under-five children in rural parts of the country. Gender of children showed that the under-five death was meaningfully fewer for females, compared to the male counterparts (HR = 0.728 95% CI: 0.606-0.875, *P* = 0.001), and this indicates that the female gender had a reduced risk of death by 27.2%, compared to male gender of the child (Table 4).

**Table 3:** Test of proportional hazards assumption

**Variables**	**rho**	**Chi- Square**	**df**	***P*** ** value**
Gender of the children	-0.03798	0.68	1	0.409
Place of delivery	0.07019	2.48	1	0.115
Family size	-0.06901	2.21	1	0.137
Mother education	-0.04081	0.89	1	0.346
Number of under-five children	0.10781	6.39	1	0.012
Contraceptive use	-0.01218	0.07	1	0.790
Source of drinking water	-0.01619	0.12	1	0.726
Global test		11.46	7	0.120

**Table 4:** Multivariable analysis result of Cox PH model of under-five year children death

**Variables**	**Estimates (SE)**	**HR (95% CI for HR)**	***P*** ** value**
Gender of the children			
Male	Ref.		
Female	-0.317 (0.094)	0.728 (0.606, 0.875)	0.001
Place of delivery			
Home	Ref.		
Health facility	-0.304 (0.129)	0.738 (0.572, 0.951)	0.019
Other places	-0.268 (0.382)	0.765 (0.361, 1.619)	0.483
Family size			
1-3	Ref.		
≥ 4	0.228 (0.096)	1.256(1.040, 1.517)	0.018
Mother education			
Not educated	Ref.		
Primary	-0.042 (0.118)	0.959 (0.761, 1.208)	0.720
Secondary and above	-0.768 (0.220)	0.464 (0.301, 0.714)	0.000
Number of under-five children			
No	Ref.		
1-2	-0.531(0.187)	0.588 (0.408, 0.849)	0.005
≥ 3	0.550 (0.223)	1.733 (1.120, 2.682)	0.014
Contraceptive method use			
No	Ref.		
Yes	-0.227 (0.167)	0.797 (0.662, 0.960)	0.017
Source of drinking water			
Piped	Ref.		
Others	0.265 (0.113)	1.303 (1.044, 1.627)	0.041

 Moreover, children who were delivered in the health facility had a reduced under-five mortality rate, compared to those delivered at home holding other variables constant (HR = 0.738; 95% CI: 0.572-0.951, *P* = 0.019). This implies a significant association of child survival time with the place of delivery. Children who were delivered at home had 26.2% more risk of mortality than those who were born in the health center. Additionally, family size is found to have a significant effect on under-five children’s mortality. An estimated hazard ratio of children whose family had three and above family size (HR = 1.256; 95% CI: 1.040-1.517, *P* = 0.018) implies that children from families having three and above family size are 1.256 times more likely to experience death than their counterparts (1-3 children). Furthermore, children from a mother with secondary school and above educational background had a reduced risk of death, compared to those from uneducated mothers (HR = 0.464; 95% CI: 0.301-0.714, *P* = 0.001). This result disclosed that children who were born from mothers with secondary school and above educational experience had 53.6% higher survival time, compared to those from uneducated mothers (Table 4).

 The risk of death among under-five children having three and above under-five brothers and sisters in the household was 1.733 times higher than their counterparts (HR = 1.733; 95% CI: 1.120-2.682, *P* = 0.014), and these children had a 73.3% higher risk of death, compared to those having no other under-five children.

 The estimated hazard ratio for children from mothers who practiced using contraceptive methods at a different time (HR = 0.797; 95% CI: 0.662-0.960, *P* = 0.017) showed that mothers who experienced using contraceptive methods had a decreased influence of under-five mortality, compared to women having no experience of any methods of contraception. The children from households not having piped water sources for drinking had an increased risk of death, compared to children from families of having piped water (HR = 1.303; 95% CI: 1.044-1.627, *P* = 0.041), and it is indicated that children from households having no piped drinking water sources were 1.303 times more likely to die than children from households of having piped water in rural parts of the country (Table 4).

## Discussion

 There is still a high proportion of under-five children’s death globally with the highest burden in the rural areas of developing countries, including Ethiopia. This study aimed to examine the risk factors associated with the mortality of under-five children in rural parts of Ethiopia using the survival analysis method. Out of the under-five children involved in this study, 6.5% of the cases died before their fifth birthday, and the results of this study are in line with the findings of a study conducted in Northern Ghana (6.14%).^21^ However, it was very low, compared to the results of a previous study performed in Ethiopia using EDHS data of 2011 (18.3%).^16^ This could imply the decreasing ratio of child death in the preceding five years of the survey time of the country.

 Out of the total children who died, 59.7% of the cases were male, and it is higher, compared to the reports of a previously conducted study in Ethiopia (50.27%)^16^; however, it is in line with the results of a study performed in the rural areas of Northern Ghana (53.3%).^21^ The higher rate in this study could be due to the variation of the study sites (rural versus urban). However, this study still implies a higher mortality rate of children in rural parts of the country. Of all children under study, most of them (77.2%) were given birth at home among whom the death proportion was very high (83%) indicating the impact of giving birth at home, and this is consistent with the results of other studies in Ethiopia^15^ and Southern Ghana.^21^ This can be considered a good indicator for most of the mothers not attending the ANC visit properly, and this could be due to the absence of health facilities that provide ANC in their localities.

 In this study, to analyze the risk factors of under-five death, non-parametric and semi-parametric regressions were applied. Gender of the child, place of delivery, family size, mother’s educational status, number of under-five children, use of contraceptive methods, and sources of drinking water were among the identified factors of under-five children’s death in the country. In this regard, female children had a reduced hazard of death than males, and it is in line with the results of several studies conducted in the Sub-Saharan region using a multi-country analysis of under-five mortality,^9^ Ethiopia,^22,23^ Ghana,^24^ and with estimates established by the UN Inter-Agency Group for child mortality estimation.^1^

 Moreover, the delivery place was meaningfully correlated with under-five deaths, and death rate of children delivered in the health facility was fewer, compared to children delivered at home. This outcome is consistent with the previous reports from sub-Saharan region,^9^ Ethiopia,^15^ Tigray regional state (qualitative study) of Ethiopia,^25^ and rural parts of Southern Tanzania.^26^ This might be because of the unavailability of enough health facilities in the nearby sites or their distant situation that might result in transportation problems.

 The household size was found to be a significant determinant of under-five death; accordingly, it is predictable that following the increase in the number of household members will increase the under-five death rate. The hazard of death for children from a household size of 4 and above is 25.6% higher, compared to children from a household of 1-3. This finding is supported by prior studies conducted in Ethiopia.^10,16^ Many researchers suggested the significant association of mothers’ educational level with under-five children’s mortality. This study also revealed that children from mothers having secondary school and above educational background had a fewer risk of death than those from uneducated ones. This is in agreement with previous studies conducted in Gilgel-Gibe Field Research Center of Southwest Ethiopia,^27^ the whole Ethiopia,^16,23,28^ Ghana,^21,24,29^ and countries of Sub-Saharan Africa.^30^

 A fewer number of under-five children in the family had a proportionally reduced death rate. This finding implied that children from mothers of having three and above under-five children in the household were 73.3% times more exposed to death, compared to other under-five children with a fewer number of sisters and/or brothers. This is in line with the findings of two studies conducted in the Sub-Saharan region^30^ and Ghana.^31^

 Mothers who were reported to use different contraceptives had significantly reduced risk of children’s mortality, compared to mothers who did not use any types of contraceptive methods to plan their family size. This is also in line with the results of studies conducted in Ethiopia^15^ and Ghana.^24^ Drinking water had found to be significantly associated with the death of under-five children. As shown in this study, children from families who did not use piped water for drinking had a higher risk of death than children from families that used piped water. This is also reported in previous studies investigated in Ethiopia^16^ and Ghana.^24^ Nevertheless, it is very contradicting with other prior studies conducted in Ethiopia.^15^ This may be due to the variation of samples that this study targeted both urban and rural residing under-five children.

## Conclusion

 This finding identified the factors that related to under-five children’s mortality in the rural parts of Ethiopia using the EDHS 2016. Applying Cox PH model variables, such as female gender, child’s birth in health facilities, having a mother with secondary school and above educational background, presence of 1-2 under-five children in the household, and having mothers who practiced contraceptive method, had reduced the risk of under-five death. However, children from 4 and above family size, 3 and above several under-five children, and birth from a family that did not use piped water sources for drinking had a higher hazard of under-five death in rural parts of the country. To overcome the death rate of children, all mothers should be aware of the factors that determine the survival times of children, all concerned bodies have to give due emphasis to the rural children since still, the mortality rate of children and mothers were high in the rural parts of different countries, including Ethiopia.

## Acknowledgment

 The authors of this manuscript would like to thank EDHS teams for these nice data collected from all parts of the country and for sending authorization letter to us, to generate and continue this study using the data set from their database. Finally, the scholars whose papers are cited in these articles and the publisher of this journal are acknowledged.

## Authors’ contributions

 Lema Abate conceived the research idea, designed the methodology, conducted analysis, and drafted the manuscript. Samuel Getachew reviewed and edited the manuscript. Both authors contributed to the interpretation and discussion of the study findings and agreed with the findings presented in the paper. Both authors have read and approved the final manuscript.

## Availability of data and materials

 Data are available in https://www.dhsprogram.com/data/dataset_admin/login_main.cfm DHS database and the extracted datasets are available from the corresponding author on reasonable request.

## Conflict of interest

 The authors declare no conflict of interest.

## Consent to participate

 Not applicable (The secondary data were taken from the DHS database).

## Ethics approval

 Ethical clearance for this study was obtained from Ethiopian Health and Nutrition Research Institute (EHNRI) Review Board, the National Research Ethics Review Committee (NRERC) at the Ministry of Science and Technology, the Institutional Review Board of ICF International, and the communicable disease control (CDC). The authors requested access to the data from the DHS program team and access was granted for the use of the data.

## Funding

 Not applicable.

**Highlights**

- About 6.5% of children were passed away before reaching their fifth-year birthday.
- The median survival time of under-five children was 31 months.
- Parental education level had a strong association with under-five survival time.
- Children delivered in health facilities had higher survival times.
- Consumption of piped water increased child survival time.
